# Compound Fracture of the Superior Maxillary Bone

**Published:** 1887-01

**Authors:** E. R. McMullen

**Affiliations:** Double Springs, Mississippi


### Compound Fracture of the Superior Maxillary Bone.

Editor Medical Brief:—I was called in haste to see a young mulatto woman who had sustained an injury of the face by being thrown from a wagon. Upon my arrival (which was four hours after the injury was received), I found my patient very much frightened, profuse hemorrhage from the mouth and anterior nares. Arresting the hemorrhage and partially arousing my patient I proceeded to make an examination. The first thing that attracted my attention was an external wound just one inch to the left of the ala of the nose on the left side. I opened her mouth with difficulty, as she had her hand wedged in her mouth to "hold my jaw up," as she told me, and when her

* Whitcomb *vs.* Reid, 31 Miss., 567.

hand was withdrawn I perceived that her jaw did drop down at least one-half of an inch, that is, the left superior maxillary bone. She had fallen on the left side of her face striking a stump three inches high; she said that she felt her jaw give way when she fell on the stump. The right superior maxillary had received no wound and was immobile, but the left was perfectly mobile, so as to feel like a "loose tooth." In the median line the left maxilla was separated from its fellow for at least two lines—one incisor being loose; in viewing the palate of the mouth I found a black line in an oblique direction from the antorior palatine foramina backward, indicating the line of fracture backward. I endeavored to elicit crepitation at other points but failed. I don't think the bone was separated from its articulation with the molars or that there could have been any fracture at or near the zygomatic surface or anywhere, the site of the buccinator for that part of the bone could not be moved and the molars were not loosened.

Now, gentlemen, crepitation, mobility, and displacement gave me a fracture of the superior maxillary bone—a compound fracture, the symptoms were evident and the diagnosis manifest. The next thing was the treatment—this was paramount to all. Here I was, just emanated from my *Alma Mater*, diploma in hand—a clinic—a compound fracture of the superior maxillary, the rarest of all fractures—in a negro hovel at midnight, a fair representation of Ethiopia as spectators. I shall not describe my armamentarium as that will best be known to those who have been in like hovels.

Thinking it best to wait till morning I gave one-half grain morphia and left the patient. Next morning found her face greatly tumefied and ecchymosis visible. I sent and got a silver wire and passed it several times around the incisors of the corresponding bones, drawing the separated bones into apposition and and lifting up the fallen jaw and maintained by means of the wire and bandages. The wire remained *in situ* for some time and kept the teeth in close relation to each other. Now, gentlemen, this was my treatment and all I knew what to do. My patient made good recovery without any deformity, although she says she cannot chew on that side as well as on the other side, and slight paralysis in the muscles of the cheek give rise to a kind of peculiar expression on the affected side.

I am going to ask many friends, who perchance will read this, the following questions, and they were what actuated me to write the case:

1. Will this fracture completely unite by osseous union?

2. Will she ever have perfect use of the jaw to perform the office of mastication?

3. What is the best treatment in this class of fractures?

Erichsen's last edition barely gives space to this fracture as he claims they seldom occur (see page 545, Erichsen's System and Art of Surgery, Vol. I).

I hope I have made myself plain, pardon my digression, the discipline of this journal is titled BRIEF. I trust that some one will be so kind as to wield their pen to post some friend who may be in such attitude as I, and *your* patient may not be a negro but the *elite* of society where relief, success, and reputation are at stake.

E. R. McMULLEN, M.D.

Double Springs, Mississippi.

